# P-384. Discontinuation Patterns and Virologic Failure Among Persons with HIV Receiving Long-Acting Injectable Cabotegravir-Rilpivirine Antiretroviral Therapy

**DOI:** 10.1093/ofid/ofaf695.602

**Published:** 2026-01-11

**Authors:** Sara M Hockney, Dana Mueller, Alexander Philbrick, Shannon Galvin, Jenna Berlet

**Affiliations:** Northwestern University Feinberg School of Medicine, Chicago, IL; McGaw Medical Center of Northwestern University, Chicago, Illinois; Northwestern Memorial Hospital, Chicago, Illinois; Northwestern University Feinberg School of Medicine, Chicago, IL; Northwestern Memorial Hospital, Chicago, Illinois

## Abstract

**Background:**

Long-acting injectable cabotegravir-rilpivirine (LA CAB/RPV) is a novel antiretroviral treatment (ART) option for virologically suppressed persons with HIV who have no prior treatment failure and no known or suspected resistance to either agent. However, in practice, patients often initiate LA CAB/RPV with incomplete or unknown treatment and resistance histories. This study aimed to characterize reasons for LA CAB/RPV therapy discontinuation in clinical practice and to describe a subset of cases in which virologic failure occurred.

**Methods:**

We retrospectively reviewed all patients seen in infectious diseases clinic between January 1, 2022, and April 17, 2025, who were on LA CAB/RPV for HIV treatment. Virologic failure was defined as two or more consecutive viral load measurements ≥ 200 copies/mL. Statistics were performed in R version 4.4.2.

**Results:**

During the study period, 201 PWH were treated with LA CAB/RPV for an average of 472 days (range 0-1241 d). Those on LA CAB/RPV were predominantly male (85.6%) with a mean age of 47 years. Therapy was discontinued in 41 (20%) patients. Reasons for discontinuation included transfer of care (n=11, 27%), insurance (n=8, 20%), adherence (n=7, 17%), patient preference (n=7, 17%), virologic failure (n=4, 10%), intolerance (n=3, 7%), and death (n=1, 2%). Combined genotype/phenotype testing was done at the time of failure in the four patients who discontinued therapy due to virologic failure. Two had rilpivirine resistance, one had integrase resistance, and one had both rilpivirne resistance and intermediate cabotegravir resistance. One additional patient experienced transient virologic failure with a maximum viral load of 1230 copies/mL. Resistance testing was negative, and virological suppression was subsequently achieved with no change in therapy. There was no difference in BMI between patients who experienced virologic failure and those who did not (32.06 kg/m^2^ vs 28.92 kg/m^2^, p=0.11).

**Conclusion:**

In PWH on LA CAB/RPV, discontinuation occurred primarily due to transfer of care and insurance barriers. Virologic failure was rare and was associated with underlying resistance but not BMI. These findings highlight the tolerability of LA CAB/RPV in clinical practice and the need to address access issues to optimize patient outcomes.

**Disclosures:**

Shannon Galvin, MD, GSK: Grant/Research Support

